# From Theory to Practice: The Impact of Team-Based Learning on Medical Students’ Communication Skills

**DOI:** 10.5334/pme.1595

**Published:** 2025-02-21

**Authors:** Elda Maria Stafuzza Gonçalves Pires, Stephanie N. E. Meeuwissen, Hans H. C. M. Savelberg

**Affiliations:** 1 School of Health Professions Education, Faculty of Health, Medicine and Life Sciences, Maastricht University, Maastricht, Netherlands; 2 School of Medicine, Faculdade Israelita de Ciências da Saúde Albert Einstein (FICSAE), São Paulo, Brazil; 3 Maastricht University Medical Center, the Netherlands; 4 School of Health Professions Education, Faculty of Health, Medicine and Life Sciences, Maastricht University, Maastricht, the Netherlands; 5 Evolving Academic Education, School of Health Professions Education, Faculty of Health, Medicine and Life Sciences, Maastricht University, Maastricht, the Netherlands

## Abstract

**Introduction:**

Developing communication skills is essential for medical students to work in modern medical practice. A collaborative learning environment, such as Team-Based Learning (TBL), is a promising environment for developing communication skills. In this study, we investigated 1) how medical students self-report their communication skills in a TBL environment and 2) to what extent students perceive a TBL environment as contributing to their communication skills development.

**Method:**

We conducted a quantitative study with a qualitative element involving Brazilian undergraduate medical students from one Medical School. Participants completed the Interpersonal Communication Competence Scale and the Team-Based Learning Environment Scale, including an open-ended question. We used ANOVA to compare responses across the seven semesters and thematic analysis for the open-ended responses.

**Results:**

Of the 416 students invited, 307 (74%) responded to both scales. Students had high scores on communication skills. Students highly valued the contribution of five domains of the TBL learning environment: teachers’ decisions, teachers’ attitudes, students’ characteristics, team characteristics, and contextual factors. The sixth domain, formative assessment, was highly valued by first-year students with a downward trend across semesters. Key factors contributing to communication skills development were teachers’ alignment with the educational methodology and students’ attitudes within teams. Additionally, students noted that their perception of safety and trust to provide feedback influenced their communication skills development.

**Conclusion:**

These findings suggest that a TBL environment can maintain students’ communication skills. Various elements of the TBL environment play a role here, particularly teachers’ alignment, students’ attitudes, and a supportive classroom atmosphere.

## Introduction

Interpersonal communication skills are the ability to effectively exchange information between individuals, express themselves, and interpret others’ verbal and non-verbal communication codes [1]. Such skills are essential for medical students to succeed in interprofessional practice [2]. Traditionally, communication skills are taught through lectures, seminars, simulations, and small group workshops using role-plays [3]. However, Gilligan et al. showed that these interventions may lead to only modest short-term improvements in students’ communication skills, with unclear long-term benefits [3]. Collaborative learning environments have been suggested as a more effective alternative for developing communication skills [4,5,6].

Collaborative learning environments are instructional settings that encourage cooperative interactions and shared learning experiences, shifting from individual learning to group-based tasks. These settings aim to create a synergistic atmosphere where students actively engage with course content, learn from one another, and develop critical thinking and problem-solving skills [4,6,7,8,9]. Team-Based Learning (TBL) exemplifies this approach, where students interact and learn from each other through group activities [5,10,11]. Individual and group readiness assurance tests are used to check whether students have understood the basic concepts of the pre-class material [5,11]. Through group problem-solving activities, students apply the course concepts. Students are expected to develop in-depth knowledge, problem-solving, and communication skills through interaction with peers while solving problems and disagreements [5,11]. TBL also involves periodical peer assessment on readiness, punctuality, communication, and collaboration [5].

**Figure 1:**
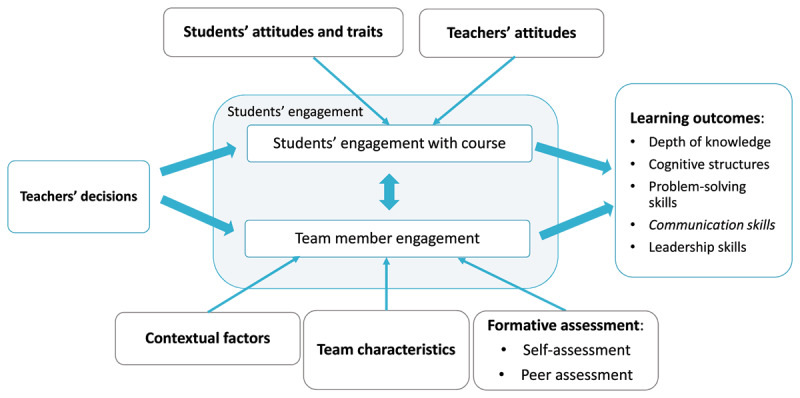
Conceptual Model of Team-Based Learning based on Michaelsen et al. [5]. Formative assessment was added to the model, and the learning outcome, ‘communication skills,’ was the primary learning outcome of this study.

Many elements in the TBL learning approach can influence learning, specifically interpersonal communication skills development. For example, teachers’ decisions about how they design the session, employing key TBL elements, may influence how students engage with each other and the course content. Other mediating factors, as found by Michaelsen et al. [5], are teachers’ attitudes, students’ attitudes and traits, team characteristics, and context factors. Teachers’ attitudes refer to teachers’ enthusiasm for the topic and satisfaction with their teaching. Students’ attitudes and traits refer to how much they value discipline, their motivation to pursue a good grade in the course, and their prior experience and academic abilities. Team characteristics relate to the mix of personalities in the team, leadership within teams, and the amount of value students place on working in teams. Lastly, contextual factors refer to the amount and regularity of contact hours during the course, acoustics and comfort of the classroom, what is simultaneously and sequentially going on in the other courses, and the school’s culture towards learning [5]. Additionally, in collaborative learning environments with TBL, students are supposed to learn from interaction with their peers and peer assessment, an essential component of classic TBL [5]. As Gilligan et al. concluded in their review, feedback is an important strategy to improve communication skills [3]. Therefore, receiving feedback through the TBL peer assessment process may also serve as a tool for students to enhance their communication skills [11]. This is captured in Figure 1, based on the model by Michaelsen et al. [5].

Studies present mixed findings regarding the benefits of TBL in developing communication skills, and its long-term effects remain unexplored [12,13,14,15]. Eun and Young found a significant improvement in nursing students’ communication skills when TBL was combined with simulation compared to the simulation-alone control group [12]. Lee showed that nursing students improved their perceptions of communication skills after only a few TBL sessions, whereas the lecture-based control group showed no improvement [13]. Conversely, Lochner et al. reported that 25 hours of interprofessional TBL during an anatomy course did not enhance communication, teamwork, or interprofessional learning [14]. Despite the widespread implementation of TBL to promote meaningful learning and developing communication skills, no research has been conducted to measure medical students’ communication skills in a TBL environment and how this environment contributes to communication skills development. Therefore, this study aimed to understand 1) how medical students self-report their interpersonal communication skills in a TBL environment and 2) to what extent students perceive a TBL environment as contributing to their communication skills development.

## Materials and Methods

### Design

We designed a primarily quantitative study with a qualitative element to explore the extent of students’ interpersonal communication skills and the factors influencing students’ development of communication skills in a collaborative learning environment [16]. With this approach, quantitative and qualitative data were collected simultaneously, analysed independently, and then compared.

### Setting

This study was conducted at the School of Medicine of Faculdade Israelita de Ciências da Saúde Albert Einstein (FICSAE) in São Paulo, Brazil. Since its establishment in 2016, the six-year undergraduate medical programme has employed collaborative learning across all subjects. Applicants are selected based on their performance in a written exam and multiple mini-interviews [17].

The programme’s first seven semesters cover basic sciences and pre-clinical subjects through collaborative, group problem-solving activities. Lectures are not used. Seven to nine subjects co-occur each semester. TBL sessions occur twice or thrice weekly following the classic TBL design [5]. All TBL sessions start with pre-class preparation, in which students must study the pre-assigned material provided by the teacher. Then, in-class TBL sessions begin with a readiness assurance test (RAT) to check whether students have studied the pre-class material and understood the basic concepts [5]. The test is done individually (iRAT) and then repeated in teams (tRAT) so students can help each other understand the basic concepts. The second part of the TBL session involves applying the course concepts with team problem-solving activities where students need to explain their reasoning to peers, relate ideas, and integrate information [11,15,18]. Teachers are advised to design problems based on the 4S principles of TBL, meaning that students must work on the same and significant problems, select a specific answer, and report their answers simultaneously, using letter cards [5,11,18,19]. Each class has 60 students divided into nine teams, with six to seven students each. Teams are assigned by the faculty, with the goal of ensuring gender diversity. These teams work together through all subjects for one semester. This structure allows for extended engagement within the team over the course of the semester: the program includes 26 hours of contact time per week, totalling an average of 520 hours of collaborative activities per semester, with 50 of those hours dedicated to TBL. At the start of each semester, teams are reshuffled.

In addition, FICSAE has a formative assessment programme, where students self- and peer-assess their team members three times per semester using an online system. The assessment is focused on readiness for classes, participation, contribution during discussions, respect and flexibility, communication skills, ability to provide and receive feedback, and professionalism. A 5-point Likert scale is used with frequency anchors (never, sometimes, half the time, often, always), and narrative feedback is required. Teachers are encouraged to provide written feedback through the online system. For each class, one teacher is responsible for compiling all the information and providing written feedback for each student three times per semester. These teachers also give verbal feedback to each student once a semester.

### Participants

In May 2023, after three months of classes, we invited all 416 students from the first seven semesters of the medical undergraduate program (from the first to the fourth academic year) to participate in the study. About one-third of the students are male, and two-thirds are female. After a TBL session, the first author (EMSGP) explained the research purpose in each class, in which students were invited to answer an online survey once. A total of 318 students participated (76%), with 63% female and 37% male participants.

### Data collection

The survey included the Interpersonal Communication Competence Scale and the Team-Based Learning Environment Scale. Data were collected using REDCap, an online survey manager [20,21].

The first section of the survey utilised the Interpersonal Communication Competence Scale (ICSS) to measure communication skills. ICCS was initially developed by Rubin and Martin [1] and validated to measure interpersonal communication skills and assess skill achievement over time. The validated survey in Portuguese [22] (see Appendix 1) has 17 items distributed over five domains (Environmental Control, Self-Disclosure, Assertiveness, Interaction Management, and Immediacy) with a Cronbach’s α of 0.82 [22]. Students were asked to answer this survey based on their interpersonal communication with their peers. Responses were recorded on a 5-point Likert scale with anchors for each answer. The total score ranges from 17 to 85, with higher scores indicating stronger interpersonal communication competencies.

The second section of the survey used the Team-Based Learning Environment Scale (see Appendix 2). This scale was designed by the research team to measure students’ perceptions of the influence of different elements of the learning environment on their communication skills development. The 14 scale items were based on the conceptual TBL model [5] (see Figure 1), which suggests that various learning environment elements influence student engagement with course content and the team, leading to different learning outcomes like communication skills. The items addressed six domains: teachers’ decisions, teachers’ attitudes, students’ attitudes and traits, team characteristics, contextual factors, and formative assessment (self and peer assessment). Five domains were extracted from the TBL model, while formative assessment was added, given the importance of peer assessment in classic TBL and its potential to influence communication skills development. The items clearly defined the TBL concepts and described how they could influence communication skills development. Students responded on a 5-point Likert scale that ranged from (1) “not at all true of me” to (5) “very true of me”. To better understand students’ perceptions of the main elements of the TBL environment that contribute to or hinder their communication skills development, the survey closed with an open-ended question: “Are there any more elements of this learning environment that contribute to or hinder the development of your interpersonal communication competence?”. The survey was piloted with medical graduates from FICSAE, leading to minor modifications for clarity.

### Data analysis

All data were anonymised before the analysis took place. The mean scores for the ICCS and the TBL environment domains were calculated per semester, and comparisons between the seven semesters were made using analysis of variance (ANOVA). Internal consistency was assessed using Cronbach’s α and McDonald’s ω, with ICCS scores of 0.82 and 0.83, respectively, and TBL Environment Scale scores of 0.80 and 0.83, respectively.

Two researchers (EMSGP, SNEM) conducted a thematic analysis of the anonymised answers to the open question [23]. They independently coded the data deductively, using codes based on the TBL model [5] (identifying the first two themes) and, inductively, identifying a new element (the third theme). Next, they discussed codes, using their various perspectives and experiences, until agreement was reached. Three overarching themes were then constructed through discussions with the entire research team.

### Reflexivity

The first author (EMSGP) is the Vice Dean for Academic Affairs at FICSAE’s School of Medicine. She collected data after explaining the research purpose and the anonymisation process to students in each class. Despite the power difference between students and the first author, the institution’s culture promotes continuous improvement, and students are encouraged to be honest and provide feedback on what works and what does not in evaluations. By offering an online, anonymous, and voluntary possibility to participate, we aimed to mitigate students’ reluctance to express their opinions openly.

The other researchers have vast experience in research in health professions education. While analysing the anonymised transcripts and discussing themes, the research team kept an open mind, moving from an analytical to a conceptual level. They discussed disagreements using their perspectives, expertise, and experiences to accurately portray students’ views on the learning environment.

### Ethical considerations

Participation was voluntary, and it was emphasised that it would not affect students’ academic activities. All participants gave informed consent prior to participation. Anonymisation was applied. This study was approved by the Ethics and Research Committee of Hospital Israelita Albert Einstein on May 10^th^, 2023, under n^o^ 5545–23. Students aged 18 or over who agreed to participate in the research signed the online Informed Consent form through REDCap [20,21].

## Results

Of the 416 students invited, 318 (76%) signed the informed consent and answered the Interpersonal Communication Competence Scale (ICCS). Eleven of these students did not complete the second section of the survey, which led to 307 (74%) students answering the ICCS and the Team-Based Learning Environment Scale. Seventy-six (18%) students answered the open-ended question (see Table 1).

**Table 1:** Interpersonal Communication Competence Scale (ICCS) and Team-Based Learning Environment Scale’s answers rate and ICCS scores per semester. ICCS scores range from 17 to 85.


SEMESTER OF THE COURSE	NUMBER OF INVITED STUDENTS	NUMBER OF ICCS ANSWERS (ANSWER RATE)	NUMBER OF TEAM-BASED LEARNING ENVIRONMENT SCALE ANSWERS (ANSWER RATE)	NUMBER OF OPEN-ENDED QUESTION ANSWERS (ANSWER RATE)	ICCS MEAN SCORE (SD)

1st	61	60 (98%)	60 (98%)	16 (25%)	66.5 (8.9)

2nd	62	51 (82%)	51 (82%)	13 (20%)	64.9 (8.9)

3rd	62	52 (84%)	45 (73%)	8 (13%)	65.0 (9.4)

4th	55	33 (60%)	33 (60%)	10 (18%)	65.9 (7.4)

5th	56	52 (93%)	52 (93%)	5 (9%)	66.6 (6.1)

6th	62	35 (56%)	33 (53%)	9 (14%)	66.0 (8.6)

7th	58	35 (60%)	33 (57%)	15 (26%)	66.7 (8.3)

Total	416	318 (76%)	307 (74%)	76 (18%)	65.9 (8.3)


### How do medical students self-report their interpersonal communication skills in a TBL environment?

Overall, students had high scores in ICCS, with a mean of 65.9 (SD 8.3). Scores ranged from 37 to 83, with means ranging from 64.9 (SD 8.9) to 66.7 (SD 8.3) over the seven semesters (see Table 1). There was no significant difference in ICCS scores between the semesters, *F*(6, 311) = 0.396, *p* = 0.881. The mean scores for each of the five domains, Environmental Control, Self-Disclosure, Assertiveness, Interaction Management, and Immediacy, did not show any trend of differences over semesters. Considering the low Cronbach’s α in each domain [22], no comparison was made between the five domains over the seven semesters.

### To what extent do students perceive a TBL environment as contributing to their communication skills development?

**Figure 2:**
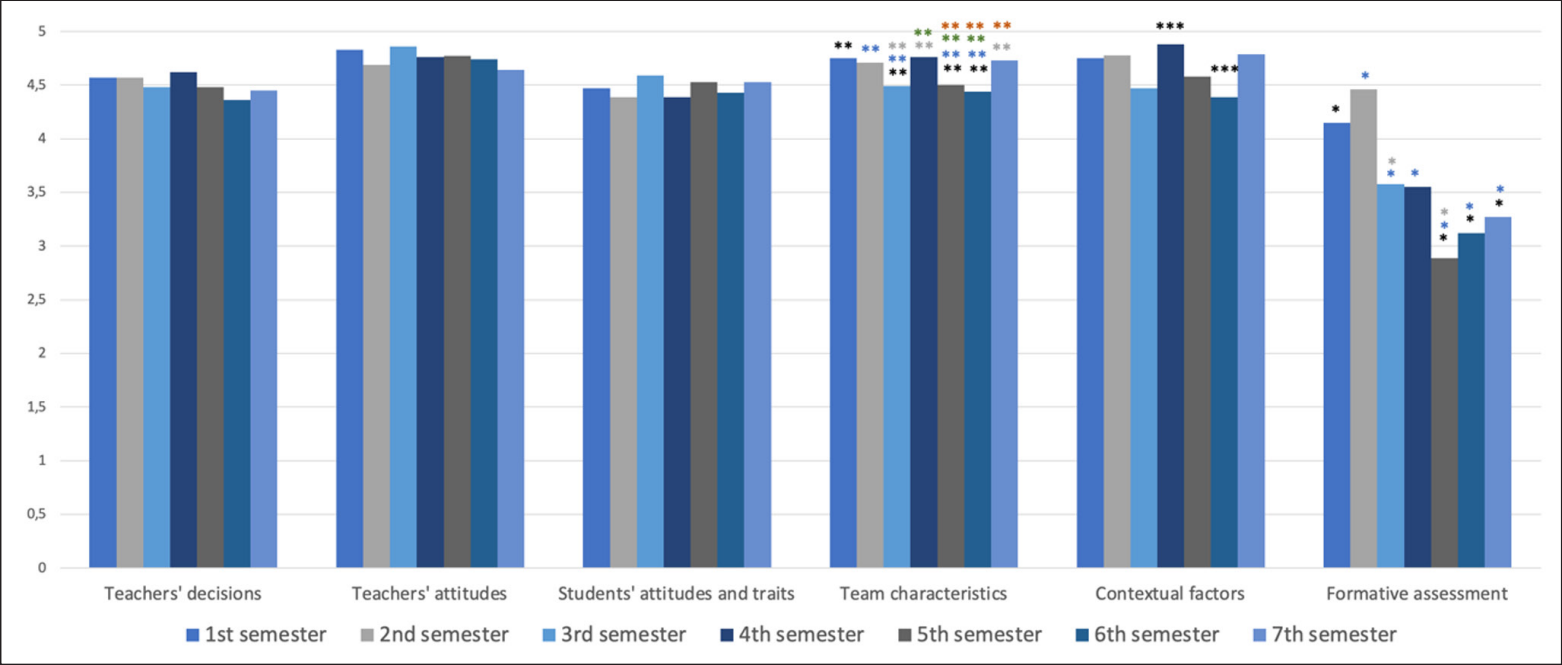
Means of each domain of the Team-Based Learning Environment Scale for each semester of the course. * Shows significant differences: For formative assessment, (*) students from the first semester scored this element significantly higher than those from the fifth, sixth semester, and seventh semesters. (*) Students from the second semester scored this element significantly higher than those from the third, fourth, fifth, sixth, and seventh semesters. (*) Students from the third semester scored this element significantly higher than those from the fifth semester. For team characteristics, (**) students in the first semester scored this element significantly higher than students in the third, fifth, and sixth semesters. (**) Students in the second semester scored this element significantly higher than students in the third, fifth, and sixth semesters. (**) Students in the third semester scored this element significantly lower than students in the fourth and seventh semesters. (**) Students from the fourth semester scored this element significantly higher than students in the fifth and sixth semesters. (**) Students in the fifth and sixth semesters scored this element lower than students in the seventh semester. For contextual factors, (***) students in the fourth semester scored this element significantly higher than in the sixth semester.

Students reported that five out of the six domains of the TBL environment contributed to developing their communication skills. Specifically, teachers’ decisions, teachers’ attitudes, students’ attitudes and traits, team characteristics, and contextual factors all scored means above 4.3 throughout the semesters. In contrast, the sixth domain, formative assessment (including both self- and peer-assessment), had an overall mean score of 3.6 (SD 0.5) (see Figure 2). Significant differences were found between the seven semesters in three TBL environment domains: team characteristics, contextual factors, and formative assessment. For team characteristics, *F*(6, 300) = 5.03, *p* = 0.002, and contextual factors, *F*(6,300) = 7.76, *p* = 0.006, there was no clear trend over semesters. However, for formative assessment, *F*(6,299) = 92.7, *p* < 0.001, a downward trend was noted, with first-year students (first and second semester) rating this domain higher compared to students from the third to seventh semester (see Figure 2).

The analysis of the 76 students’ responses to the open-ended question revealed three main themes that explain how the TBL elements contributed to students’ communication skills development: teachers’ alignment with the educational methodology, students’ attitudes in the teams, and students’ perception of safety and trust to provide feedback. The first two themes reinforced the Team-Based Learning Environment Scale findings. The third theme introduced a new element of the collaborative learning environment that impacted students’ communication skills development.

#### Teachers’ Alignment with the Educational Methodology

Students revealed that the design and execution of classes by teachers influenced both the quantity and quality of team discussions, which in turn affected their communication skills development. On the one hand, students noticed that if teachers did not use collaborative learning methods or failed to design suitable cases that promoted team discussions, their communication skills development was hindered. On the other hand, if a case was designed with adequate complexity and discussion time, students wrote that they engaged in the discussion, which was essential for developing communication skills. As one participant wrote:

*“When the cases are well prepared to stimulate discussion within the team, without requiring knowledge that we do not yet have or that is too easy [the case discussion contributes to my communication skills development].”* (Participant 158)

Additionally, the dynamic way the teachers conducted the class helped to maintain student engagement. Participants outlined that when teachers made it possible to discuss cases among multiple teams, this, too, significantly facilitated their communication development. One student remarked:

*“[The opportunity to] discuss cases between teams, rather than just within our team, significantly helps [communication skills development]. Defending the team’s point [of view] (not necessarily your own) strengthens my discussion skills”*. (Participant 74)

#### Students’ Attitudes in the Teams

Students indicated that their attitudes within teams could influence the quality of the discussions and, consequently, their communication skills development. When students felt engaged to work as a team, discussions were more productive, which enhanced their communication skills development. Students also shared how they learned to interact and adapt their interpersonal communication skills when team members encountered personal issues:

*“For example, the unpredictability of life for each team member leads us to problematic situations, which requires great communication skills to comfort, demand, and motivate.”* (Participant 19)

Most students were optimistic about changing teams every semester, as explained by the following participant:

*“Working every semester with different people is tiring, but it is gratifying when you realise how much it helps handle interpersonal conversations.”* (Participant 305)

However, some students mentioned how team discussions were hindered when team members had different motivations for learning or lacked a sense of affinity with each other. This was described by one participant:

*“I believe that as much as the constant change of members helps to develop skills for dealing with people with whom we have no affinity, a lack of affinity between the members diminishes the discussion and generates stress that might otherwise not exist.”* (Participant 166)

In some teams, students admitted that they avoided conflict resolution since they were scared off by other students’ attitudes or lacked confidence in their skills. Without productive team discussions, further communication skills development was hindered, as one student described the influence of students’ attitudes:

*“I think it depends on the group I’m in. If the members are more welcoming, I feel free to communicate. But if more arrogant people are in the group, I become inhibited and find it more difficult to express myself.” (Participant 142)*

#### Students’ Perception of Safety and Trust to Provide Feedback

From students’ perspectives, the perception of safety and trust influenced their communication skills development since it impacted the quality of peer assessment. Depending on the feedback quality, peer assessment was valued as a potential contributing element to communication skills development. On the one hand, students noted how peer assessment positively influenced communication skills development since constructive feedback allowed reflection on what they needed to improve:

*“The TBL environment fosters a feedback culture, which contributes to developing our communication skills. In my opinion, not only is my improvement in this competence visible, but also that [improvement of communication skills] of several classmates”*. (Participant 310)

On the other hand, many students wrote that their peers did not take peer assessment seriously, and they did not provide constructive feedback to each other. Without specific, constructive feedback, they could not optimally improve their communication skills:

*“There is a certain fear of formative [peer] assessment, so I realise that many people aren’t honest to avoid intrigue in the team and gossip. I’ve heard many people complaining behind other’s backs when someone in their team commented something negative on the formative assessment”*. (Participant 41)

## Discussion

This study aimed to understand how medical students self-report their interpersonal communication skills in a TBL environment and to what extent students perceive a TBL environment as contributing to their communication skills development. We found that students achieved high Interpersonal Communication Competence scores in this collaborative Team-Based Learning environment. Participants highly valued the contribution of five key domains of the TBL environment for their communication skills development: teachers’ decisions, teachers’ attitudes, students’ characteristics, team characteristics, and contextual factors [5]. However, the sixth domain, formative assessment, was highly valued by first-year students but less so by more senior students, as revealed by a downward trend in appreciation across semesters. Furthermore, we recognised two main elements of the collaborative learning environment that were considered crucial for communication skills development: teachers’ alignment with the educational methodology and students’ attitudes in the teams. Additionally, students noticed that their perception of safety and trust to provide feedback influenced their communication skills development.

The consistently high ICCS scores could indicate that students in this learning environment are stimulated to maintain their communication skills across the semesters. Team discussions during tRAT and problem-solving activities are the main moments when students interact while solving problems and disagreements. While interacting, explaining, and justifying their choices, students may develop their assertiveness, interaction management, and immediacy, which are important domains of interpersonal communication skills [1,5,22]. For first-semester students, these findings may reflect the effect of intensive collaborative learning during the initial three months of the course, combined with multiple mini-interviews (MMIs) in the admission process [17]. Since communication skills are assessed during MMIs, incoming students may have already possessed strong communication skills. The absence of differences across semesters could be attributed to the high level of communication skills from the programme’s start and the supportive nature of the collaborative learning environment, which may help students maintain these high scores on ICCS. Communication skills in a TBL environment have been measured previously in nursing students [12]; however, our study makes a unique contribution to the literature by assessing medical students’ communication skills in a TBL environment, where they engage in an average of 50 hours of TBL per semester. Moreover, this study was conducted in a well-established curriculum that has employed TBL for almost a decade across the first seven semesters of the programme. Therefore, our findings provide insights into the long-term effect of TBL on communication skills, addressing some of the questions raised in Reimschisel et al.’ s systematic review [15]. Another important finding was that students highly valued the contribution of the five key domains of the TBL environment to their communication skills development. Additionally, students emphasised that teachers’ alignment with the educational methodology and students’ attitudes within the teams were important factors influencing their communication skills development. These findings align with the TBL conceptual model and our previous study, which identified the challenge posed by teachers and commitment to the group as the main drivers for students to take a deep approach to learning [8]. Teachers’ and students’ attitudes are crucial for achieving the proposed learning outcomes in a student-centred collaborative learning environment [8,11,18]. Our findings underscore the importance of teachers maintaining the structure of TBL as initially designed and building upon the initial model by Michaelsen et al. [5,18,24]. To enhance communication skills, teachers can contribute by ensuring students have the appropriate prior knowledge, creating cases that stimulate discussions, and allocating time for discussions in and between teams [6,9,11,19].

Students also valued students’ attitudes within the teams and the practice of changing teams every semester. Since teams need time to collaborate effectively [10,11,25], spending more time together could optimise group dynamics, trust and cohesion [11]. We found that changing groups every semester allowed students to work with different students, diversifying their experiences and presenting meaningful opportunities for communication skills development. This finding aligns with previous recommendations in TBL literature to intentionally form diverse teams rather than based on affinity [5,11,19] and let the team “stay together for as long as possible” [10,11]. Educational organisations might consider applying these findings by keeping teams consistent across various subjects within a semester while reorganizing them at the start of each new semester to preserve the advantages of team diversity.

A surprising finding of this study was the downward trend regarding the perceived importance of formative assessment (self and peer assessment) for students’ communication skills development across semesters. Since Gilligan et al. highlighted feedback as an important strategy to improve communication skills [3], we anticipated peer assessment to be highly valued. While some students appreciated peer assessment when it was specific and constructive, others either did not take it seriously or failed to provide constructive feedback. Interestingly, this finding mirrors earlier research in TBL environments, which showed that students may not consistently or honestly provide constructive feedback [26,27,28,29,30]. Students manipulate their peer’s ratings to satisfy the grading requirements [26] and provide high scores to not harm one another [27]. When looking at the quality of the comments, most students focus on positive aspects [28,29] and feel reluctant to point out gaps and/or suggestions for further improvement [28]. Our results suggest that some students did not feel safe enough to provide honest feedback or did not see peer assessment as fundamental for developing communication skills. However, we could not explore the reasons behind the downward trend across semesters in-depth because we collected qualitative and quantitative data simultaneously in this research. Therefore, future research is needed to explore how students perceive safety and trust to provide feedback in a TBL learning environment. Understanding students’ beliefs about peer assessment and exploring how students engage and provide actual peer feedback will be crucial for improving the learning environment and optimising the effectiveness of peer assessment.

There are some limitations to this study. First, the study was done in only one institution. Next, its cross-sectional design precludes evaluating how communication skills developed over time. Third, since communication skills were measured through a self-assessment questionnaire, a social desirability bias may exist, where students responded with socially acceptable reactions instead of being honest. However, participants rated some items poorly, showing that they did not respond in line with perceived expectations only. Lastly, as we collected quantitative and qualitative data simultaneously, we could not determine why students from the third to seventh semesters valued formative assessments less than first-year students. Notwithstanding, our pioneering study fills a critical gap in the literature by exploring the various elements of the TBL learning environment, deepening our understanding of how key elements of the TBL environment contribute to communication skills development [15].

Our findings suggest that a collaborative learning environment incorporating TBL can maintain students’ communication skills. While we could not show a difference in the communication skills of students experiencing TBL for more than three years compared to students experiencing TBL for three months, all students showed high scores of ICCS and perceived this learning environment as beneficial for developing their communication skills. Educational organisations might consider reviewing and revising their lecture-based undergraduate medical programmes and implementing more collaborative learning activities [4,9,10,11,31]. When adopting TBL, it is important for teachers to maintain the original structure of the approach to foster rich team discussions [9,11,18,24]. Additionally, we recommend assigning students to teams for extended periods to optimise interaction, team cohesion and communication skills development [6,11,25]. Educational institutions must help students become aware of how team dynamics and students’ attitudes can impact communication skills development. This is essential to maximise learning experiences. Feedback through peer assessment could be of tremendous value in this context [5,11]. However, further research is needed to explore how to improve the learning environment to support students in providing and receiving constructive peer assessment.

## Additional Files

The additional files for this article can be found as follows:

10.5334/pme.1595.s1 Appendix 1. Interpersonal Communication Competence Scale (ICCS).

10.5334/pme.1595.s2 Appendix 2. Team-Based Learning Environment Scale.
