# Supplementary material for: From Theory to Practice: The Impact of Team-Based Learning on Medical Students’ Communication Skills
Source: Perspect Med Educ. 2025 Feb 21;14(1):74–84. doi: 10.5334/pme.1595 (PMC11843980; doi:10.5334/pme.1595)
Supplement: Appendix 1. — Interpersonal Communication Competence Scale (ICCS). [file pme-14-1-1595-s1.pdf]

Appendix 1 - Interpersonal Communication Competence Scale (ICCS) [1,22]

INSTRUCTIONS: Here are some statements about how people interact with each other. For each statement, circle the response that best reflects YOUR communication with others. Be honest in your answers and think very carefully about your communication behaviour. Mark only one alternative in each item. Do not leave any questions blank.

The interaction that will be analysed is between classmates.

If you almost always interact in this way, circle 5.

If you usually communicate in this way, circle 4.

If you sometimes behave this way, circle 3.

If you rarely interact like this, circle 2.

If you almost never behave this way, circle 1.

1 I stand up for my rights.	1	2	3	4	5
2 In conversations with classmates, I perceive not only what they say, but also what they do not say.	1	2	3	4	5
3 I can persuade others to my position.	1	2	3	4	5
4 I reveal how I feel to others.	1	2	3	4	5
5 I take charge of conversations I'm in by negotiating what topics we talk about.	1	2	3	4	5
6 I tell people when I feel close to them.	1	2	3	4	5
7 I accomplish my communication goals.	1	2	3	4	5
8 I have trouble standing up for myself.	1	2	3	4	5
9 I let others know that I understand what they say.	1	2	3	4	5
10 My classmates truly believe that I care about them.	1	2	3	4	5
11 I allow my classmates to see who I really am.	1	2	3	4	5
12 Others would describe me as warm.	1	2	3	4	5
13 I express myself well verbally.	1	2	3	4	5
14 I try to look others in the eye when I speak with them.	1	2	3	4	5
15 When I've been wronged, I confront the person who wronged me.	1	2	3	4	5
16 Other people think that I understand them.	1	2	3	4	5
17 It is difficult to find the right words to express myself.	1	2	3	4	5

Interpersonal Communication Competence Scale (ICCS) translated and validated in Brazilian Portuguese [22]

Escala de Competência em Comunicação Interpessoal

INSTRUÇÕES: aqui estão algumas afirmações sobre como as pessoas interagem entre si. Para cada afirmação, circule a resposta que melhor reflete SUA comunicação com os outros. Seja honesto em suas respostas e reflita, com muito cuidado, sobre o seu comportamento de comunicação. Marque só uma alternativa em cada item. Não deixe nenhuma questão em branco.

Responda o questionário baseando-se na sua relação com seus colegas de classe.

Se você quase sempre interage desta maneira, circule 5.
Se você geralmente se comunica desta maneira, circule 4.
Se você às vezes se comporta desta maneira, circule 3.
Se você interage assim raramente, circule 2.
Se você quase nunca se comporta desta maneira, circule 1.

1 Defendo meus direitos.	1	2	3	4	5
2 Em conversas com colegas de sala, percebo não apenas o que eles dizem, mas o que não dizem.	1	2	3	4	5
3 Consigo persuadir os outros quanto à minha opinião.	1	2	3	4	5
4 Revelo como me sinto para os outros.	1	2	3	4	5
5 Assumo o controle das conversas em que estou envolvido, negociando os tópicos sobre os quais falaremos.	1	2	3	4	5
6 Digo às pessoas quando me sinto próxima delas.	1	2	3	4	5
7 Atinjo meus objetivos de comunicação.	1	2	3	4	5
8 Tenho dificuldade em me defender.	1	2	3	4	5
9 Deixo que os outros saibam que compreendo o que eles dizem.	1	2	3	4	5
10 Meus colegas realmente acreditam que me preocupo com eles.	1	2	3	4	5
11 Permito que os meus colegas vejam quem realmente sou.	1	2	3	4	5
12 Outros me descreveriam como caloroso(a), ou seja, afetuoso(a).	1	2	3	4	5
13 Expresso-me bem verbalmente.	1	2	3	4	5
14 Tento olhar os outros nos olhos quando falo com eles.	1	2	3	4	5
15 Quando sou injustiçado(a), confronto a pessoa que me injustiçou.	1	2	3	4	5
16 Outras pessoas acham que eu as entendo.	1	2	3	4	5
17 É difícil encontrar as palavras certas para me expressar.	1	2	3	4	5
