# Supplementary material for: From Theory to Practice: The Impact of Team-Based Learning on Medical Students’ Communication Skills
Source: Perspect Med Educ. 2025 Feb 21;14(1):74–84. doi: 10.5334/pme.1595 (PMC11843980; doi:10.5334/pme.1595)
Supplement: Appendix 2. — Team-Based Learning Environment Scale. [file pme-14-1-1595-s2.pdf]

Appendix 2 – Team-Based Learning Environment Scale

This questionnaire has several sentences about the elements of the learning environment and how they may influence the development of your interpersonal communication competence with your classmates.

There is no *right* way of answering it. It is, accordingly, important that you answer each question as honestly as you can. If you think your answer to a sentence would depend on the subject being studied, give the answer that would apply to the subject(s) most important to you.

Please choose the appropriate option alongside the question number. The answers range from 1: This item is *not at all true* of me to 5: This item is *very* true of me.

Please choose the most appropriate response to each question. Select the answer that best fits your immediate reaction. Do not spend a long time on each item: your first reaction is probably the best. Please answer each item.

Refrain from projecting a good image. Your answers are CONFIDENTIAL. I appreciate your cooperation.

Competencies regarding interpersonal communication are defined as the skill individuals have to exchange information between two or more people, expressing themselves and interpreting others' communication codes (verbal or non-verbal)

1. Participating in all team phases of TBL (tRAT, case discussions) helps me to improve my interpersonal communication competence.
2. When teachers design cases that challenge us, it helps me to improve my interpersonal communication competence.
3. Teachers' enthusiasm for the topic helps me to develop my interpersonal communication competence.
4. When teachers listen to students without judgment and create a safe learning environment, I feel engaged to improve my interpersonal communication competence.
5. When I think the subject is more important, I am engaged to improve my interpersonal communication competence.
6. When I think pursuing a good grade in the subject is important, I feel engaged to improve my interpersonal communication competence.
7. My prior experience working in teams before medical school helped me develop my interpersonal communication competence.
8. Being in a team with classmates with different personalities helps me to develop my interpersonal communication competence.
9. TBL teams' self-organising character helps me develop my interpersonal communication competence.
10. TBL is a good learning strategy to develop my interpersonal communication competence.
11. Changing teams every semester helps me to develop my interpersonal communication competence.
12. Having class adapted to TBL sessions, where I can sit with my team for all the disciplines, helps me to develop my interpersonal communication competence.
13. The exercise of self-assessment helps me to develop my interpersonal communication competence.
14. Peer assessment helps me to develop my interpersonal communication competence.

Final open question:

15. Are there any more elements of this learning environment that contribute to or hinder the development of your interpersonal communication competence?

Team-Based Learning Environment Scale in Brazilian Portuguese

Este questionário contém uma série de frases sobre os elementos do ambiente de aprendizagem e como eles podem influenciar o desenvolvimento de sua competência de comunicação interpessoal com seus colegas de classe.

Não há uma maneira certa de respondê-lo. Portanto, é importante que você responda a cada pergunta da forma mais honesta possível. Se você acha que sua resposta a uma frase dependeria do assunto que está sendo estudado, dê a resposta que se aplica ao(s) assunto(s) mais importante(s) para você.

Escolha a opção apropriada ao lado do número da pergunta. As respostas variam de 1: este item não é totalmente verdadeiro para mim a 5: este item é muito verdadeiro para mim.

Por favor, escolha a resposta mais adequada para cada pergunta. Selecione a resposta que melhor se adapta à sua reação imediata. Não gaste muito tempo em cada item: sua primeira reação é provavelmente a melhor. Por favor, responda a cada item.

Não se preocupe em projetar uma boa imagem. Suas respostas são CONFIDENCIAIS.

Competência de comunicação interpessoal é definida como a habilidade que os indivíduos possuem para trocar informações entre duas ou mais pessoas, expressando-se e interpretando os códigos de comunicação dos outros (verbal ou não verbal)

1. Participar de todas as fases de discussão em grupo do TBL (tRAT, casos) me ajuda a melhorar minha competência de comunicação interpessoal.
2. Quando os professores elaboram casos que nos desafiam, isso me ajuda a melhorar minha competência de comunicação interpessoal.
3. O entusiasmo dos professores pelo tópico da aula me ajuda a desenvolver minha competência de comunicação interpessoal.
4. Quando os professores ouvem os alunos sem julgamento e criam um ambiente de aprendizagem seguro, sinto-me empenhado(a) em melhorar minha competência de comunicação interpessoal.
5. Quando acho que o assunto é mais importante, sinto-me empenhado(a) em melhorar minha competência de comunicação interpessoal.
6. Quando acho importante obter uma boa nota na disciplina, sinto-me engajado(a) a aprimorar minha competência de comunicação interpessoal.
7. Minha experiência anterior, trabalhando em grupos antes da Faculdade de Medicina, me ajuda a desenvolver minha competência de comunicação interpessoal.
8. Participar de um grupo com colegas com diferentes personalidades me ajuda a desenvolver minha competência de comunicação interpessoal.
9. O fato de cada grupo de TBL poder se organizar do seu jeito me ajuda a desenvolver minha competência de comunicação interpessoal.
10. Acredito que o TBL é uma boa estratégia de aprendizado para desenvolver minha competência de comunicação interpessoal.
11. Mudar de grupo a cada semestre me ajuda a desenvolver minha competência de comunicação interpessoal.
12. Ter salas de aula adaptadas para TBL, onde posso sentar com o meu grupo em todas as disciplinas, me ajuda a desenvolver a minha competência de comunicação interpessoal.
13. A autoavaliação periódica (da avaliação formativa) me ajuda a desenvolver minha competência de comunicação interpessoal.
14. A avaliação de pares me ajuda a desenvolver a minha competência de comunicação interpessoal.

Pergunta aberta:

15. Existem mais elementos deste ambiente de aprendizagem do TBL que contribuem ou atrapalham o desenvolvimento de sua competência de comunicação interpessoal?
